# Hormonal contraceptive use is associated with reduced central serotonergic activity indexed by the loudness dependence of auditory evoked potentials

**DOI:** 10.3389/fnhum.2025.1647425

**Published:** 2025-10-01

**Authors:** Henrik Normannseth, Christoffer Hatlestad-Hall, Trine Waage Rygvold, Alena Hadzic, Stein Andersson

**Affiliations:** ^1^Section for Clinical and Cognitive Neuroscience, Department of Psychology, University of Oslo, Oslo, Norway; ^2^Department of Neurology, Oslo University Hospital, Oslo, Norway; ^3^Department of Clinical Neurosciences for Children, Oslo University Hospital, Oslo, Norway; ^4^Section for Pharmacology and Pharmaceutical Biosciences, Department of Pharmacy, University of Oslo, Oslo, Norway; ^5^Section of Psychosomatic Medicine, Division of Mental Health and Addiction, Oslo University Hospital, Oslo, Norway

**Keywords:** hormonal contraceptives, sex hormones, loudness dependence of auditory evoked potential (LDAEP), central serotonergic activity, depressive symptoms

## Abstract

**Objective:**

Hormonal contraceptives (HCs) are linked to mood disturbances, but the neurobiological mechanisms remain unclear. This study investigated whether HC use is associated with altered central serotonergic activity, using the loudness dependence of auditory evoked potentials (LDAEP).

**Methods:**

Fifty-four healthy women (30 current HC users and 24 non-users) completed EEG recordings to assess LDAEP. Depressive symptoms were quantified using the Beck Depression Inventory-II. Between-group analyses were controlled for age and depressive symptoms, and effects of menstrual cycle phase, HC type, and mood-related side effects were also examined.

**Results:**

HC users showed significantly steeper LDAEP slopes than non-users across components (all *p* ≤ 0.028) consistent with reduced central serotonergic activity. This remained significant controlling for age and depressive symptoms. No significant effects of menstrual cycle phase or HC type, but HC users reporting adverse mood effects had more somatic symptoms, without corresponding LDAEP differences.

**Conclusion:**

This study is, to our knowledge, among the first to explicitly test an *a priori* hypothesis that HC use is associated with reduced serotonergic activity indexed by LDAEP in healthy women, and shows that HC use is linked to attenuated central serotonergic activity independent of mood symptoms. These findings underscore the role of sex hormones in shaping serotonergic function and may explain individual variability in mood and antidepressant response.

## 1 Introduction

Depression is a leading global health concern, disproportionately affecting women, particularly during their reproductive years (World Health Organization, 2023). While numerous biological and psychosocial factors may contribute to this disparity, fluctuations in sex hormones (estrogen and progesterone) have been identified as crucial triggers of negative mood changes in women (Albert, 2015; Kuehner, 2017). Hormonal contraceptives (HCs), used by over 400 million women worldwide (United Nations Department of Economic and Social Affairs, 2023), introduce synthetic sex hormones (estrogen and progestin) which modulate the neuroendocrine cascade responsible for controlling the menstrual cycle (follicular, ovulation, and luteal phase) and reproduction (Levin and Hammes, 2011). Today, two types of HCs are available in pharmacies: drugs containing a combination of estrogen and progestin or drugs containing progestin-only, which can be administered through several routes. Despite the widespread use of HCs, concerns have been raised regarding their potential neuropsychiatric effects, including mood alterations and an increased risk of depressive symptoms (Larsen et al., 2020; Lewis et al., 2019; Skovlund et al., 2016). However, the neurobiological mechanisms underlying these potential side effects remain poorly understood.

Although debated, one proposed mechanism of mood disorders involves the serotonergic system, which plays a central role in emotional regulation and has been implicated in the pathophysiology of depression (Bremshey et al., 2024). Both endogenous and exogenous sex hormones influence serotonergic transmission through multiple pathways, including changes in serotonin synthesis, receptor density, and reuptake mechanisms (Gasbarri et al., 2012; Wharton et al., 2012). Estrogen has been associated with increased serotonin synthesis (Hampson, 2023) as well as up-regulation of 5-HT receptor expression and reduced MAO-A activity (Wharton et al., 2012), thereby promoting higher serotonergic tone. In contrast, progestins have been linked to reduced 5-HT receptor binding (Pletzer et al., 2023) and elevated MAO-A activity (Hampson, 2023), which may dampen serotonergic signalling. Neuroimaging studies have provided further support for this interaction, demonstrating altered 5-HT receptor availability in HC users compared to non-users (Larsen et al., 2020). Given these findings, it has been hypothesized that the use of HCs modulates serotonin-dependent brain function in ways that may influence both mood stability and response to serotonergic antidepressants (Larsen et al., 2022).

A promising electrophysiological probe for assessing central serotonergic activity is the loudness dependence of auditory evoked potentials (LDAEP), a non-invasive measure reflecting central serotonergic modulation of the auditory cortex (Hegerl and Juckel, 1993). LDAEP is derived from the N1-P2 component of auditory evoked potentials (AEPs) and is calculated as the slope of amplitude change in response to increasing auditory stimuli intensity. Serotonergic projections from the raphe nuclei to the primary auditory cortex modulate neuronal excitability, leading to an inverse relationship between LDAEP and central serotonergic activity (Juckel et al., 1999). A steeper LDAEP slope (greater change in amplitude with increasing stimulus intensity) suggests lower central serotonergic activity, whereas a shallower LDAEP slope indicates higher central serotonergic function (Hegerl et al., 2001). Experimental evidence supports this mechanistic link, pharmacologically elevating brain serotonin in cats suppresses LDAEP magnitude (Juckel et al., 1997), and acute SSRI administration in placebo-controlled crossover studies consistently flattens the LDAEP magnitude in healthy adults (Nathan et al., 2006; Simmons et al., 2011). LDAEP has been studied in psychiatric populations, particularly in depression, where it has been identified as a predictor of treatment response to selective serotonin reuptake inhibitors (SSRIs; Hegerl et al., 2001; Yoon et al., 2021). However, despite its clinical relevance, research on LDAEP in healthy populations, particularly in the context of HC use and mood regulation, is largely lacking.

Previous studies have reported sex differences in LDAEP, with women exhibiting stronger LDAEP slopes than men, suggesting lower central serotonergic activity (Oliva et al., 2011). Additionally, preliminary findings suggest that healthy HC users display stronger LDAEP responses compared to natural menstrual cycling women, potentially reflecting further serotonergic modulation due to synthetic sex hormone exposure (Andersson et al., 2025). Given the established link between serotonergic dysregulation and mood disturbances, further investigation into how HC use may influence serotonergic function is warranted. HC formulations differ in estrogen content, progestin type, dose, and route of administration, which can yield different systemic exposure profiles (Sitruk-Ware and Nath, 2013). Estrogenic components may support serotonergic signaling (Wharton et al., 2012), whereas progestins have been linked to reduced serotonergic tone through receptor-level and monoaminergic mechanisms (Pletzer et al., 2023). Because all contemporary HCs deliver a synthetic progestin and modify endogenous ovarian hormone rhythms, we treated HC use as a class-level exposure *a priori* and examined formulation-specific effects exploratorily.

This study aimed to examine whether healthy women currently using HC exhibit differences in central serotonergic activity, as indexed by the LDAEP, compared to non-users. A secondary objective was to explore whether LDAEP magnitudes correlate with self-reported depressive symptoms, thereby providing insight into potential neurobiological mechanisms linking HC use to mood disorder vulnerability. To our knowledge, this is among the first studies to explicitly test an *a priori* hypothesis that HC use is associated with reduced serotonergic activity as indexed by LDAEP in a non-clinical female sample, extending earlier exploratory findings (e.g., Andersson et al., 2025).

## 2 Materials and methods

### 2.1 Participants

A total of 54 healthy females (30 HC users and 24 non-users; mean age = 25.7, *SD* = 4.4 years) were recruited through flyers posted at the University of Oslo, Oslo, Norway. Among current HC users, 17 (56.7%) used combined HCs, while 13 (43.3%) used progestin-only HCs.

Inclusion criteria included being aged between 18 and 40 years, possessing normal hearing, and currently not being pregnant or breastfeeding. Exclusion criteria included any previous or current psychiatric or neurological disorders, ongoing substance abuse, or use of psychoactive medications. Hearing was assessed through basic audiometric testing immediately before EEG recording.

All participants provided written informed consent before participation and received compensation equal to 30 US dollars for their participation. The study was approved by the Regional Ethics Committee, South-East Norway (REK ref.: 657156).

Participants were asked to indicate the reason for their use of HC, including both contraceptive and non-contraceptive indications. Among current users, 28 of 30 (93.3%) reported using HC solely to prevent pregnancy. Two participants (6.7%) reported use for menstrual pain relief (dysmenorrhea); one of these also reported a diagnosis of polycystic ovary syndrome (PCOS) and used HC as hormonal treatment for PCOS-related symptoms. No participants reported a history of endometriosis, premenstrual dysphoric disorder (PMDD), or amenorrhea. HC type (combined vs. progestin-only) was recorded, but the primary analysis pooled all current HC users to maximize power and estimate class-level effects. Participants using any hormonal contraceptive method (including oral pills, injectables, implants, and hormonal IUDs) were classified as current HC users. Age of HC initiation was recorded for both current and past users. Duration of current HC use was not collected, and cumulative exposure could therefore not be analyzed.

For participants reporting a natural menstrual cycle, cycle phase was determined by self-reported cycle day in a brief questionnaire. Phases were categorized as follicular, ovulatory, or luteal based on day ranges, consistent with prior questionnaire-based research.

### 2.2 Questionnaires

#### 2.2.1 HC usage assessment

Participants completed a questionnaire recording their history of HC use, mood effects, and general health factors. HC users provided details about their current HC type and whether they had experienced mood changes. Past HC users were asked about reasons for discontinuation and mood effects. Additional lifestyle factors, including alcohol, nicotine, and medication use, were recorded to control for potential confounds.

#### 2.2.2 Beck Depression Inventory-II (BDI-II)

The Beck Depression Inventory-II (BDI-II; Beck et al., 1996) was used to assess depressive symptoms over the previous 2 weeks. This 21-item questionnaire has a total score range of 0–63, with higher scores indicating greater symptom burden. A cut-off score of 13 was used to differentiate between above-threshold and below-threshold depressive symptoms, in line with prior research (von Glischinski et al., 2019). BDI-II scores were analyzed using the cognitive and somatic-affective subscales defined by Dozois et al. (1998), based on the female-specific factor structure derived in their sex-separated analysis.

### 2.3 EEG/LDAEP methodology

#### 2.3.1 Recording protocol and setup

The LDAEP paradigm was presented after an initial 8 min of resting-state EEG, ensuring that participants had not been exposed to other auditory stimuli beforehand. Participants were seated in a dimly lit, sound-attenuated room designed to minimize external noise. Participants were instructed to maintain their gaze on a red dot displayed centrally on a 24” LCD screen (BenQ, model ID: XL2420-B) when not otherwise engaged with reading instructions or asked to close their eyes, and to minimize facial movements throughout the experiment.

Auditory stimuli consisted of 1,000 Hz tones delivered binaurally at intensities of 55, 65, 75, 85, and 95 dB, each lasting 30 ms. Each intensity level was presented 80 times in a pseudo-randomized order with an interstimulus interval (ISI) of 1,200–1800 ms. Auditory stimuli were generated using Psychtoolbox-3 (Kleiner et al., 2007) in MATLAB (version 2015a; MathWorks, Natick, MA, USA), presented via an Alto AMX-80 mixing console (inMusic Brands, Inc., Cumberland, RI, United States) and conveyed through Etymotic ER-1 insert earphones (Etymotic Research, Inc.).

#### 2.3.2 EEG data acquisition and preprocessing

EEG signals were recorded using a 64-channel BioSemi ActiveTwo system with electrodes placed in accordance with the international 10–10 system (Oostenveld and Praamstra, 2001). Ocular activity was recorded via four additional electrodes placed laterally and vertically around the eyes to facilitate artifact rejection. EEG data were sampled at 2048 Hz with an online high-pass filter at 0.16 Hz and an anti-aliasing filter.

Preprocessing was conducted using functions from the EEGLAB framework for MATLAB (Delorme and Makeig, 2004), in four stages. First, the raw data were organized in accordance with the Brain Imaging Data Structure (BIDS; Pernet et al., 2019), re-referenced to the average of all EEG channels, and, to facilitate computational efficiency without compromising temporal resolution significantly, EEG signals were re-sampled to 512 Hz. Second, each participant’s EEG data were assessed for noisy channels using an automated pipeline. For the bad channel detection, the signals were temporarily high-pass filtered at 1 Hz and line noise (50 Hz) was suppressed using spectral interpolation (Leske and Dalal, 2019). Channels were evaluated based on their variance, inter-correlation with other channels, channel noise captured by independent component analysis, and power spectra. Third, after excluding bad channels and re-referencing to the average of the remaining channels, bad segments and ocular artifacts were removed from the data using automated procedures. After band-pass filtering between 1 and 100 Hz, and suppressing 50 Hz line noise, bad segments were identified as 5-s blocks of data where more than 25% of the channels exceeded a conservative amplitude standard deviation (25 μV). Then, independent components reflecting ocular and muscular artifacts were subtracted from the signal. Independent component analysis was implemented using the Second-Order Blind Identification (SOBI) algorithm (Belouchrani et al., 1993), while component classification was done using the ICLabel toolbox (Pion-Tonachini et al., 2019). Fourth, after applying a 30 Hz low-pass filter, the EEG was segmented into 600 ms epochs, each containing one stimulus event (−100 ms pre-stimulus onset to 500 ms post-stimulus onset). Noisy epochs were identified using the FASTER toolbox default threshold (Nolan et al., 2010) and discarded. The mean number of epochs per participant were 76.3, 76.6, 76.4, 76.1, 76.3 for the 55 dB, 65 dB, 75 dB, 85 dB, and 95 dB conditions, respectively. There was no significant effect of condition on the number of available epochs.

### 2.4 LDAEP calculation

The LDAEP was calculated from the N1, P2, and N1P2 peak-to-peak amplitudes at central midline electrode, Cz, based on the averaged AEP waveforms for each participant. Cz was chosen as the site for LDAEP calculation, as it has been commonly used in previous LDAEP studies (Hagenmuller et al., 2016; Hwang et al., 2021). The N1 amplitude was defined as the most negative peak occurring between 60 and 140 ms following stimulus onset, while the P2 amplitude was identified as the most positive peak within the 150–250 ms range. Peak identification was manually confirmed for all participants and conditions. For each component (N1, P2, and N1P2), LDAEP was quantified as the slope of a linear regression line fitted to amplitude values across the five stimulus intensities, reflecting the degree of amplitude modulation with increasing sound intensity. The N1P2 amplitude was calculated as the peak-to-peak difference between P2 and N1 at each intensity level, and its slope was derived from these values.

### 2.5 Statistical analyses

All analyses were conducted in R 4.5.0 (R Core Team, 2025) using base functions and the packages *effsize* (Torchiano, 2020)*, psych* (Revelle, 2025)*, car* (Fox and Weisberg, 2019), and *emmeans* (Lenth, 2025). Prior to inferential testing, variable distributions were assessed using the Kolmogorov–Smirnov test. LDAEP slope values (N1, P2, and N1P2) were approximately normally distributed within groups.

Unadjusted group differences in LDAEP slopes were evaluated using one-way analysis of variance (ANOVA). The primary analysis compared all current HC users (formulations pooled) with non-users, justified by the presence of exogenous hormones across methods. Exploratory contrasts between combined and progestin-only users were also conducted, though these subgroups were small (*n* = 17 vs. 13) and results interpreted cautiously. Although the comparison involved only two groups, ANOVA was used to align with the structure of the subsequent analysis of covariance (ANCOVA) models. Each slope was then reanalyzed using ANCOVA to adjust for age and depressive symptomatology (BDI-II somatic-affective and cognitive subscales). Analyses including depressive symptoms were pre-specified as secondary and exploratory, since participants were not recruited on the basis of mood symptoms. Differences in LDAEP slope values across menstrual cycle phases were analyzed using one-way ANOVA.

ANCOVAs were conducted using Type II sums of squares via the *car* package. Multicollinearity was assessed using variance inflation factors (VIFs), all of which were below 2.5. Adjusted group means were reported as estimated marginal means using the *emmeans* package. Effect sizes for both ANOVA and ANCOVA models were reported using partial *η*^2^, with conventional benchmarks for interpretation: small = 0.01, medium = 0.06, large = 0.14.

Categorical variables (e.g., education level, contraceptive type, menstrual phase) were compared using Pearson’s chi-square test or Fisher’s exact test, depending on expected cell sizes. Age of HC onset and depressive symptom scores were analyzed using the Wilcoxon rank-sum test due to non-normal distributions. Subgroup comparisons (e.g., adverse mood effects, contraceptive type) were conducted using independent-samples *t*-tests or Wilcoxon tests, based on distributional assumptions. All tests were two-tailed, with alpha set to 0.05.

An *a priori* power analysis was conducted using G*Power 3.1 (Faul et al., 2009), based on the sample size and effect size (*d* = 0.77) reported in Andersson et al. (2025). The analysis indicated that a total sample size of 46 participants would be required to achieve 80% power at an alpha level of 0.05.

## 3 Results

### 3.1 Descriptive group statistics

Table 1 presents descriptive statistics for age, education level, menstrual phase, age of HC usage onset, and self-reported adverse mood effects across the three initial groups: current HC users (*n* = 30), past users (*n* = 17), and never-users (*n* = 7). The groups did not significantly differ in age, *F*(2, 51) = 2.14, *p* = 0.129, or in education level, *χ^2^*(4) = 8.01, *p* = 0.091. Among participants who reported having a menstrual cycle and were aware of their cycle phase (*n* = 40), no significant group differences were found in the distribution across follicular, ovulatory, and luteal phases, *χ^2^*(4) = 1.28, *p* = 0.865. Likewise, the age of HC initiation did not significantly differ between current and past users (*W* = 280.5, *p* = 0.572, Wilcoxon rank-sum test). Duration of current HC use was not available. Three participants reported non-contraceptive indications for HC use: two for dysmenorrhea and one for PCOS. Single-case tests showed that their LDAEP slopes did not differ significantly from other HC users (all *p* ≥ 0.05). Sensitivity ANCOVAs excluding these participants confirmed that the main HC effect remained significant.

**Table 1:** Demographic and health-related characteristics across initial groups.

Baseline characteristic	Current HC users	Past users	Never-users	*p*-value
N	30	17	7	
Age	25.3 (3.9)	27.2 (5.2)	23.4 (3.4)	0.129*
Education - High school - College	13 (43.3%) 17 (56.7%)	5 (29.4%) 12 (70.6%)	4 (57.1%) 3 (42.9%)	0.091^†^
Age of HC onset	17.1 (2.7)	16.6 (1.7)	N/A	0.572^‡^
Adverse mood effects - Yes - No	13 (56.5%) 10 (43.5%)	14 (82.4%) 3 (17.6%)	N/A	0.085^†^ 0.103^§^
Menstrual cycle phase - Follicular - Ovulation - Luteal	8 (40.0%) 6 (30.0%) 6 (30.0%)	4 (28.6%) 4 (28.6%) 6 (42.9%)	2 (33.3%) 1 (16.7%) 3 (50.0%)	0.865^†^
HC types		N/A	N/A	
Combined HC (*n* = 13)				
- Oral pill	11			
- Vaginal ring or transdermal patch	2			
Progestin-only HC (*n* = 17)				
- Oral pill	4			
- Subdermal implant or depot injection	5			
- Intrauterine device	8			

^*^ = ANOVA, ^†^ = Chi-squared, ^‡^ = Mann–Whitney U, ^§^ = Fisher’s exact test.

### 3.2 Rationale for merging past users and never-users into a single group

A key objective of this study is to isolate the neurophysiological effects of ongoing HC usage. To our knowledge, there are no longitudinal studies showing whether central serotonergic markers normalize after discontinuation. Available evidence is limited to cross-sectional comparisons of current users and non-users. In this sample, all but two former HC users had ceased usage for at least 1 year. We therefore assessed comparability empirically in this dataset.

Initial analyses also indicated that past users did not differ significantly from never-users in key demographic variables (e.g., age, education) or clinical measures (including LDAEP and depressive symptoms). Omnibus ANOVAs confirmed group effects on N1 and N1P2 slopes, and *post hoc* (Tukey) comparisons showed steeper slopes in current users versus past users, while past and never users did not differ. Moreover, the never-user subgroup was notably small (*n* = 7), diminishing the statistical power and interpretive clarity for comparisons among the three groups. By merging past users and never-users into a single “non-users” group, the study achieves both a more robust sample size and a clearer contrast against current HC users. This approach enhances the statistical power to detect meaningful differences attributable to active HC usage and focuses the analysis on the immediate effects of exogenous hormones on central serotonergic activity.

### 3.3 LDAEP differences between current HC users and non-users

**Figure 1:**
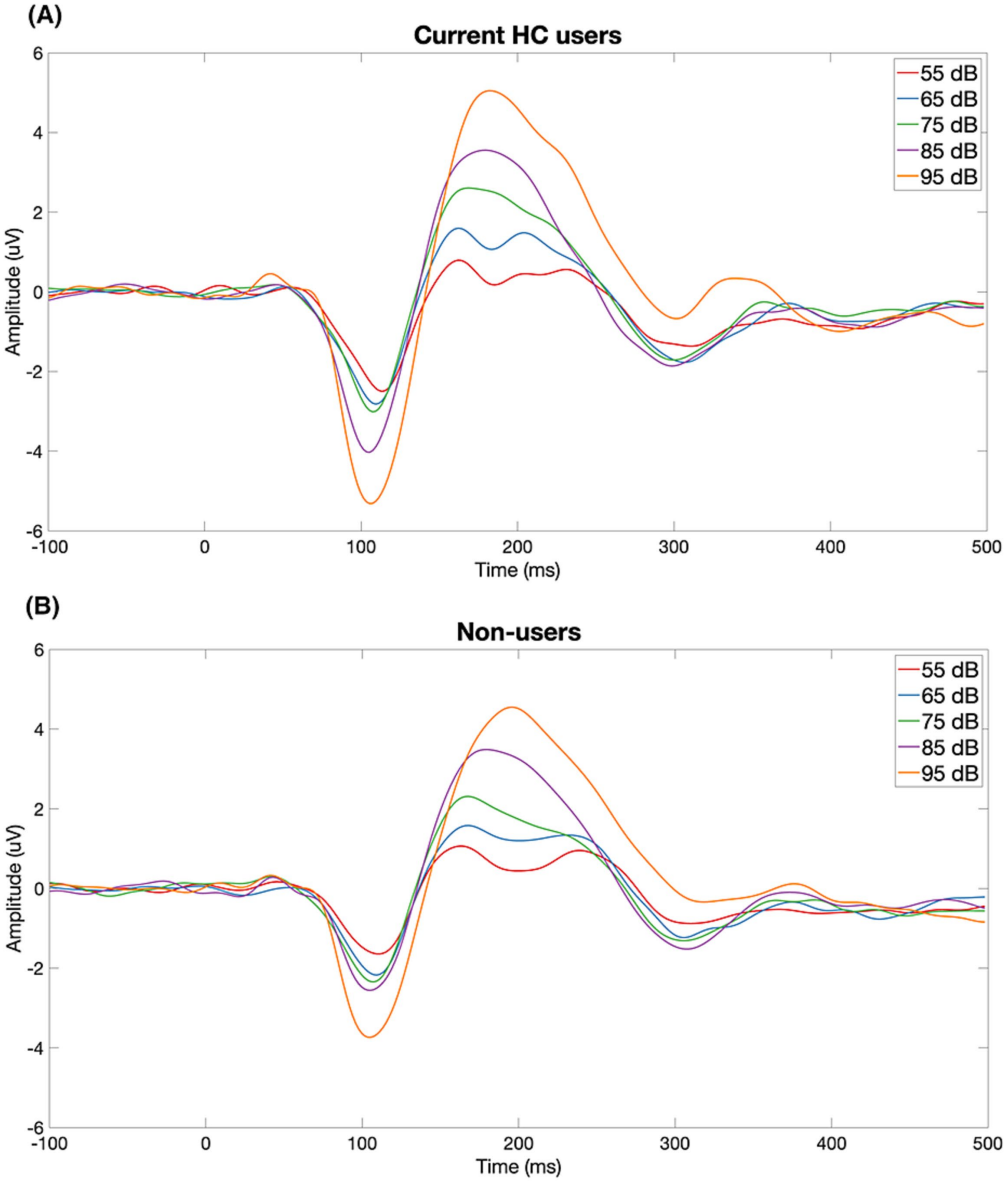
Grand-average ERP waveforms at Cz for current HC users **(A)** and non-users **(B)** in response to five stimulus intensities (55–95 dB SPL). Each trace reflects the group-averaged response to one intensity level. HC users show a visibly steeper N1–P2 amplitude change across intensities than non-users, consistent with larger LDAEP.

**Figure 2:**
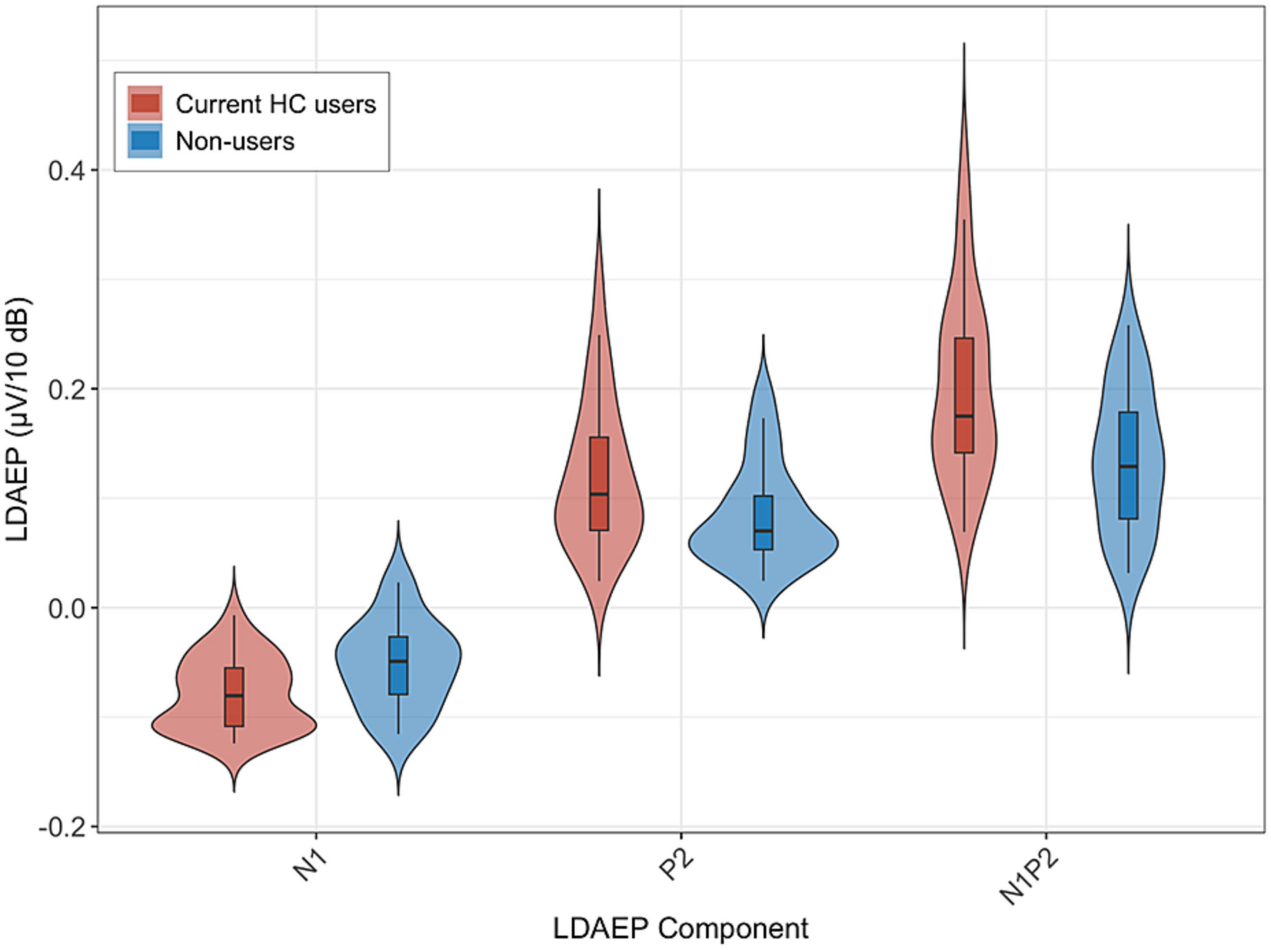
LDAEP slopes at Cz (N1, P2, N1P2) for current HC users (*n* = 30) and non-users (*n* = 24). Violin plots show the distribution of slopes; the central line indicates the median. Group means (SD) were: N1–0.080 (0.033) vs. −0.052 (0.040), P2 0.120 (0.068) vs. 0.084 (0.046), and N1P2 0.200 (0.084) vs. 0.136 (0.064), for HC users and non-users, respectively. Group differences were statistically significant: N1 *p* = 0.006, P2 *p* = 0.028, N1P2 *p* = 0.003. Higher slope values reflect lower central serotonergic activity.

Figure 1 displays the grand-average ERP waveforms at Cz for current HC users and non-users in response to each of the five stimulus intensities. LDAEP slopes (N1, P2, and N1P2), measured at Cz, differed significantly between current HC users and non-users, as illustrated in Figure 2.

For the N1 LDAEP slope, current HC users exhibited a significantly more pronounced negative slope (*M* = −0.080, *SD* = 0.033) compared to non-users (*M* = −0.052, *SD* = 0.040). This difference was statistically significant, *F*(1, 52) = 8.05, *p* = 0.006, with an associated partial *η*^2^ of 0.13 [95% CI (0.02, 1.00)]. The mean difference between groups was −0.028 [95% CI (−0.048, −0.008)].

For the P2 LDAEP slope, current HC users exhibited a steeper LDAEP slope (*M* = 0.120, *SD* = 0.068) than non-users (*M* = 0.084, *SD* = 0.046). This difference was statistically significant, *F*(1, 52) = 5.15, *p* = 0.028, with an associated partial *η*^2^ of 0.09 [95% CI (0.01, 1.00)]. The mean difference between the groups was 0.036 [95% CI (0.004, 0.069)].

For the N1P2 LDAEP slope, current HC users exhibited a steeper slope (*M* = 0.200, *SD* = 0.084) compared to non-users (*M* = 0.136, *SD* = 0.064). This difference was statistically significant, *F*(1, 52) = 9.59, *p* = 0.003, with an associated partial *η*^2^ = 0.16 [0.03, 1.00]. The mean difference between the groups was 0.064 [95% CI (0.023, 0.106)].

### 3.4 Controlling for age and depressive symptoms

After covariate adjustment, LDAEP slopes measured at Cz remained higher in current HC users than in non-users.

N1 LDAEP slope differed significantly between current HC users and non-users after controlling for age and depressive symptom scores, *F*(1, 49) = 7.68, *p* = 0.008, partial *η*^2^ = 0.14. Neither covariate was statistically significant. Adjusted estimated marginal means were −0.080 (*SE* = 0.007) for users and −0.052 (*SE* = 0.007) for non-users. The regression model yielded *R^2^* = 0.209, adjusted *R^2^* = 0.144.

P2 LDAEP slope differed significantly between current HC users and non-users after controlling for age and depressive symptom scores, *F*(1, 49) = 5.87, *p* = 0.019, partial *η*^2^ = 0.11. Age and depressive symptoms were not significant predictors. Adjusted estimated marginal means were 0.122 (*SE* = 0.011) for users and 0.081 (*SE* = 0.012) for non-users. The regression model yielded *R^2^* = 0.116, adjusted *R^2^* = 0.044.

N1P2 LDAEP slope was significantly higher in current HC users than in non-users after controlling for age and depressive symptom scores, *F*(1, 49) = 10.13, *p* = 0.003, partial *η*^2^ = 0.17. Neither covariate showed a significant association. Adjusted estimated marginal means were 0.202 (*SE* = 0.014) for users and 0.133 (*SE* = 0.016) for non-users. The regression model yielded *R^2^* = 0.199, adjusted *R^2^* = 0.134.

### 3.5 Group differences in depressive symptoms and their association with LDAEP

As a secondary, exploratory aim, we examined whether current HC users differed from non-users in self-reported depressive symptoms by comparing BDI-II scores between the groups. Wilcoxon rank-sum tests showed no significant differences in total BDI-II, cognitive, or somatic-affective subscale scores (all *p* > 0.264).

To assess whether mild depressive symptoms modulated LDAEP, participants were stratified by a validated BDI-II threshold into subthreshold (*n* = 33) and above-threshold (*n* = 21) groups. Among current HC users, 17 were subthreshold and 13 above-threshold; among non-users, 16 were subthreshold and 8 above-threshold. Independent-samples *t*-tests comparing LDAEP slopes (N1, P2, and N1P2) between sub- and above-threshold participants across the full sample revealed no significant differences (all *p* > 0.209). Additional within-group comparisons, conducted separately for HC users and non-users, also showed no significant differences in LDAEP slopes between subthreshold and above-threshold individuals.

### 3.6 Menstrual phase and hormonal contraceptive type

Further exploratory subgroup analyses were conducted to examine whether menstrual phase or HC type was associated with LDAEP modulation. Among non-users, one-way ANOVA across self-reported menstrual cycle phases (follicular, ovulatory, and luteal) revealed no significant differences in N1, P2, or N1P2 slopes (all *p* > 0.170). Equivalent results were found in current HC users. Given that cycle phase was assessed by self-report without hormonal verification, these analyses should be considered exploratory. Additionally, among current HC users, no significant differences were observed between those using combined HCs (*n* = 17) and those using progestin-only HCs (*n* = 13) across any LDAEP component. Independent *t*-tests showed no significant group differences in N1, P2, or N1P2 slopes.

### 3.7 Self-reported adverse mood effects

Adverse mood effects attributed to HC use were reported by 11 out of 30 current HC users (36.7%) and by 14 out of 17 past users (82.4%) during their previous use. Although LDAEP slopes (N1, P2, N1P2) did not differ significantly between those with and without such reported side effects (all *p* ≥ 0.235), exploratory analyses of depressive symptoms suggested group differences. Specifically, participants who endorsed adverse mood effects scored significantly higher on total BDI-II scores (W = 158.5, *p* = 0.021) and on the somatic-affective subscale (*W* = 172, *p* = 0.004), whereas cognitive scores did not differ (*W* = 134.5, *p* = 0.201). Furthermore, a Fisher’s exact test revealed that adverse mood effects were significantly associated with being above the clinical BDI-II threshold (*p* = 0.040). Given the exploratory nature of these subgroup analyses, findings should be interpreted with caution.

## 4 Discussion

The present study investigated the effects of HC use on central serotonergic activity, as indexed by LDAEP, and its relationship with sub-clinical depressive symptoms in healthy females. The results revealed significantly steeper LDAEP slopes (N1, P2, and N1P2) among current HC users compared to non-users, suggesting lower central serotonergic activity in the former. The groups were significantly different even when controlling for age and depressive symptoms, both of which are known to influence LDAEP measures (Min et al., 2012), reinforcing the association between active HC use and altered serotonergic signaling. The strength of these effects suggests a meaningful electrophysiological distinction between current HC users and non-users. Past users did not differ from never-users on LDAEP measures, supporting their combination into a single non-user group. Neither menstrual cycle phase nor contraceptive formulation (combined vs. progestin-only) were significant predictors of LDAEP. While overall BDI-II scores did not significantly differ between users and non-users, current HC users who reported adverse mood effects scored higher on depressive symptoms, particularly somatic symptoms, though these mood effects were not reflected in altered LDAEP slopes.

The principal finding of this study is the significant elevation in LDAEP among current HC users compared to non-users. While this difference between groups was reflected in all LDAEP slopes, the effect was most pronounced for the N1P2 slope, followed by N1 and then P2. As LDAEP is widely regarded as a noninvasive proxy for central serotonergic activity, with higher values reflecting reduced serotonergic tone (Hegerl et al., 2001; Hegerl and Juckel, 1993), these results support the interpretation that active HC use is associated with diminished central serotonergic function. Notably, these effects emerged despite no significant group differences in age, education level, or menstrual cycle phase awareness, strengthening the notion that the observed alterations are linked to sex hormone exposure rather than confounding demographic or menstrual cycle-related factors. These findings may provide an electrophysiological context for large-scale epidemiological studies reporting increased depression risk in HC users (Johansson et al., 2023; Skovlund et al., 2016, 2018).

The reduced central serotonergic activity observed in current HC users may partly reflect disruptions to the neuroendocrine system associated with synthetic sex hormones. Although the degree of endogenous hormone suppression likely varies across HC formulations, recent PET evidence shows that HC users differ from non-users in central catecholamine synthesis capacity (Taylor et al., 2023). This supports the interpretation that exogenous hormones can modulate neurochemical systems regardless of formulation or systemic hormone levels. Exogenous estrogen and progestin, introduced by HC, may interfere with the body’s natural hormonal fluctuations, which play a significant role in regulating central serotonergic activity. Wharton et al. (2012) reviewed preclinical and human research indicating that estrogen generally supports serotonergic function by increasing serotonin synthesis, reuptake, receptor availability, and reducing serotonin degradation via MAO. Crucially, it may not be simply low estrogen levels influencing mood itself, but rather the suppression or alteration of endogenous hormonal rhythms induced by synthetic sex hormone use. In line with this hypothesis, Larsen et al. (2020) reported reduced 5-HT₄ receptor binding potential in HC users, consistent with diminished central serotonergic tone.

Moreover, synthetic sex hormones differ from endogenous sex hormones in terms of receptor affinity and downstream signaling, which could further influence central serotonergic balance. For example, progestins such as norethindrone have been found to reduce both progesterone and estrogen receptor expression in human tissues (Hovanessian-Larsen et al., 2012), potentially limiting the brain’s adaptive responses to hormonal signaling. Progestins might also influence serotonin metabolism by upregulating MAO activity, potentially accelerating serotonin degradation (Pletzer et al., 2023). Additionally, some progestins display estrogenic effects through direct estrogen receptor binding (Hampson, 2023), possibly introducing complexity or competition in serotonergic signaling pathways. Synthetic estrogens, such as ethinyl estradiol, also differ from natural estrogen in their receptor interactions, potentially altering central serotonergic signaling dynamics (Wharton et al., 2012). Collectively, these receptor-level alterations, combined with disrupted hormonal modulation, might contribute to the altered central serotonergic activity observed in HC users.

No significant differences in LDAEP were found between users of combined HCs and those using progestin-only HCs. This could reflect limited statistical power, as the sample sizes within each subgroup were modest. However, it may also suggest that the presence of estrogen is not the primary driver of altered central serotonergic activity. Since all users were exposed to synthetic progestins, it is plausible that progestins alone underlie the observed differences. This interpretation aligns with prior findings suggesting that progestins may exert stronger mood-related effects than estrogens (Skovlund et al., 2016), and raises the possibility that progestins, irrespective of HC type, are sufficient to modulate central serotonergic markers like LDAEP.

As expected in a healthy, non-clinical sample, depressive symptom analyses were exploratory and did not reveal significant differences between HC users and non-users, as measured by the BDI-II. Average scores in both groups were low, consistent with typical findings in non-clinical populations (Kendall et al., 1987), which may limit the scale’s sensitivity to subtle mood changes. Moreover, the restricted range of depressive symptom scores in this sample could have attenuated any association with LDAEP. Consistent with the current findings, we have previously reported no significant association between LDAEP and depressive symptoms in a healthy sample (Andersson et al., 2025), suggesting that this relationship may be weak or difficult to detect in healthy, non-clinical populations. However, other studies have found significant associations in similar cohorts. For example, Kim et al. (2016) observed that individuals with higher LDAEP values reported more depressive symptoms and greater affective lability, indicating that central serotonergic modulation may influence mood even among psychologically healthy participants. These mixed findings may stem from differences in affective variability, subclinical traits, or limited statistical power in small, low-symptomatic samples.

However, exploratory analyses revealed that current HC users, who reported adverse mood effects attributed to the HCs, scored significantly higher on both total BDI scores and the somatic subscale compared to those without such experiences. These effects were not mirrored in LDAEP slopes, suggesting that self-reported mood disturbances may arise, at least in part, independently of the serotonergic changes indexed by the current electrophysiological marker. Importantly, the lack of significant LDAEP differences between these subgroups should be interpreted with caution, as the sample sizes were relatively small and likely underpowered to detect subtle effects. The dissociation between LDAEP and mood reports in this context may therefore reflect limited statistical sensitivity rather than true absence of neurophysiological differences.

### 4.1 Future directions: hormonal contraceptive use in LDAEP and depression research

The findings of the present study highlight the importance of accounting for HC use in future research involving LDAEP. Given that HC users showed consistently elevated LDAEP slopes, indicating reduced central serotonergic activity, ignoring HC status may obscure other true group differences or introduce systematic bias. Moreover, the absence of a significant association between LDAEP and depressive symptoms in this healthy, non-clinical sample highlights the need to investigate these dynamics in clinical populations. Specifically, future studies should explore whether HC-related serotonergic alterations contribute to depressive symptomatology in vulnerable individuals or whether a subset of HC-using women with depression may exhibit a distinct serotonergic profile identifiable via LDAEP. Such work could help clarify whether HC use not only modulates central serotonergic activity but also plays a pathophysiological role in a specific subtype of depression. This line of research may ultimately enhance the precision of LDAEP as a diagnostic or predictive tool and improve the understanding of how synthetic sex hormones interact with neurobiological pathways relevant to mood disorders.

Although this study did not investigate SSRI treatment outcomes directly, the elevated LDAEP values observed among current HC users suggest central serotonergic downregulation that may have implications for antidepressant response. High LDAEP has consistently been associated with better SSRI outcomes in depressed patients (Ip et al., 2023; Yoon et al., 2021), presumably because SSRIs can effectively augment central serotonergic tone when it is initially low. However, findings from Larsen et al. (2022) complicate this relationship by showing that HC use moderates the link between central serotonergic function and SSRI efficacy. In their sample of depressed women, lower 5-HT₄ receptor binding, an index of reduced serotonergic activity, was associated with better treatment response in non-users, but with worse response in HC users. This crossover interaction implies that HC users may represent a distinct serotonergic-related subtype of depression with qualitatively different treatment trajectories. Our findings support this framework by showing that central serotonergic function, as indexed by LDAEP, is already altered in healthy HC users, suggesting that the serotonergic modulation associated with HC use may precede clinical depression. Taken together, these results underscore the need to consider HC status when interpreting central serotonergic markers like LDAEP, particularly in relation to SSRI treatment response.

### 4.2 Strengths and limitations

A key strength of this study is the demographic and clinical homogeneity of the groups, which increases internal validity by minimizing confounds. Data quality was also high, with minimal artefact rejection and use of standard EEG preprocessing pipelines. Conversely, the modest sample size may provide limited statistical power to detect marginal effects. Our primary estimand was the class-level effect of HC use, but subgroup sizes for combined vs. progestin-only users (n = 17 vs. 13) were limited, and exploratory contrasts are interpreted cautiously. The presence of one participant with PCOS and two with dysmenorrhea is another limitation, though sensitivity analyses confirmed that excluding them did not change the results. Reliance on self-reported measures (BDI-II, retrospective mood effects, menstrual cycle phase) may introduce bias and reduces precision, particularly for cycle-phase comparisons. We also lacked data on duration of current HC use; age of initiation was recorded but is only a rough proxy for cumulative exposure. Finally, heterogeneity in LDAEP acquisition and processing protocols across studies complicates direct comparisons; greater methodological standardization would aid reproducibility.

## 5 Conclusion

This study provides novel evidence that current HC use is associated with reduced central serotonergic activity, as indexed by steeper LDAEP slopes, in healthy women. These neurophysiological differences remained significant after adjusting for age and depressive symptoms and were not accounted for by menstrual cycle phase or HC type. While depressive symptoms did not differ between HC users and non-users at the group level, self-reported mood disturbances among current HC users were linked to higher BDI-II scores, particularly in the somatic domain. The absence of LDAEP differences within these subgroups may reflect insufficient power or a dissociation between subjective and neurophysiological markers of mood. Given the established relevance of central serotonergic markers such as LDAEP in predicting antidepressant response, future research should examine whether HC use interacts with treatment outcomes or contributes to distinct neurobiological profiles in depression. Taken together, these results highlight the importance of considering sex hormone exposure when interpreting central serotonergic markers in both research and clinical contexts.

## Data Availability

The raw data supporting the conclusions of this article will be made available by the authors, without undue reservation.
